# A Review of the Extraction and Determination Methods of Thirteen Essential Vitamins to the Human Body: An Update from 2010

**DOI:** 10.3390/molecules23061484

**Published:** 2018-06-19

**Authors:** Yuan Zhang, Wei-e Zhou, Jia-qing Yan, Min Liu, Yu Zhou, Xin Shen, Ying-lin Ma, Xue-song Feng, Jun Yang, Guo-hui Li

**Affiliations:** 1 Department of Pharmacy, National Cancer Center/National Clinical Research Center for Cancer/Chinese Academy of Medical Sciences and Peking Union Medical College, Beijing 100021, China; zhangyuan@cicams.ac.cn (Y.Z.); yanjiaqing5566@126.com (J.-q.Y.); liumin26081@163.com (M.L.); yz0908hospital@163.com (Y.Z.); hmuegw@126.com (X.S.); ylmacmu@163.com (Y.-l.M.); yangjun_99@126.com (J.Y.); 2 Graduate School of Peking Union Medical College, Peking Union Medical College, Chinese Academy of Medical Sciences, Beijing 100032, China; zhouweiexby@163.com; 3 School of Pharmacy, China Medical University, Shenyang 110013, China; voncedar@126.com

**Keywords:** vitamins, extraction, determination, review

## Abstract

Vitamins are a class of essential nutrients in the body; thus, they play important roles in human health. The chemicals are involved in many physiological functions and both their lack and excess can put health at risk. Therefore, the establishment of methods for monitoring vitamin concentrations in different matrices is necessary. In this review, an updated overview of the main pretreatments and determination methods that have been used since 2010 is given. Ultrasonic assisted extraction, liquid–liquid extraction, solid phase extraction and dispersive liquid–liquid microextraction are the most common pretreatment methods, while the determination methods involve chromatography methods, electrophoretic methods, microbiological assays, immunoassays, biosensors and several other methods. Different pretreatments and determination methods are discussed.

## 1. Introduction

As one of the seven major nutrients, vitamins play important roles in the body. Vitamins are involved in the processes of normal metabolism and cell regulation, and they are necessary for growth and development; thus, they are chemicals that we all need to stay healthy [1,2]. There are thirteen vitamins that are recognized as playing roles in human nutrition [3]. Based on their solubility, these vitamins can be divided into fat-soluble vitamins and water-soluble vitamins. The former contains vitamin A, D, E and K, while the latter group includes the B-complex and C vitamins.

A number of biological functions in the body have been associated with the fat-soluble vitamins [4,5,6,7,8,9,10,11,12]. Once the amount of vitamins cannot meet the body’s needs, the vitamins must be supplied from the diet. The functions and dietary sources of these fat-soluble vitamins are represented in Table 1 [13].

B-complex and C vitamins are water-soluble vitamins. The B-group is a big family, which contains B_1_ (thiamine), B_2_ (riboflavin), B_3_ (niacin), B_5_ (pantothenic acid), B_6_ (pyridoxine), B_8_ (biotin), B_9_ (folic acid), B_12_ (cyanocobalamine) and related substances. In metabolic processes, several B-group vitamins act mainly as coenzymes to produce energy and play important roles [14]. Vitamin C is one of the most important vitamins which is also indispensable for life and is involved in many important physiological processes, such as iron absorption, the immune response and so on [3]. The functions and dietary sources of these water-soluble vitamins are represented in Table 2 [13].

Different vitamins are necessary for the body to maintain normal health, as reported by the US National Institute of Health [15]. In recent years, the essential roles of vitamins in human health have received extensive attention. For people who are at risk of vitamin deficiencies, vitamin supplementation is regarded as an effective treatment (e.g., intake of multivitamin tablets). However, an overdose of vitamins can be toxic in nature [16,17,18,19,20]. In addition, interactions of vitamins and other drugs are often reported [21]. Consequently, in order to use vitamins reasonably, it is essential to develop rapid, accurate, reliable and efficient methods for the simultaneous separation and quantitation of multiple vitamins in different matrices.

Recently, research on vitamins has attracted widespread interest. The number of publications involving vitamins has increased significantly which demonstrates that these issues are becoming more and more popular. A lot of pretreatment and determination methods of vitamins were developed before 2010 [22,23,24,25,26,27,28,29,30,31,32]. At that time, general pretreatment techniques included liquid–liquid extraction (LLE), solid-phase extraction (SPE) and so on, while the determination methods include chromatography methods, electrophoretic methods and others. As we know, great progress in analytical chemistry has been achieved and some new analytical instruments have been developed since 2010. Considering few comprehensive reviews of pretreatment and determination of vitamins has been published systematically, in this paper, we presented a review of the most common sample preparation methods, including ultrasonic assisted extraction (UAE), supercritical fluid extraction (SFE), SPE, LLE, dispersive liquid–liquid microextraction (DLLME) and different analysis methods, including chromatography methods, electrophoretic methods, microbiological assays, immunoassays, biosensors and others which have been reported and used to analyze vitamins since 2010. The pretreatment methods used are summarized in Table 3 [33,34,35,36,37,38,39,40,41,42,43,44,45,46,47,48,49,50,51,52,53,54,55,56,57,58,59,60,61,62,63,64,65,66,67,68,69,70,71,72,73,74,75,76,77,78,79,80,81,82,83,84,85,86,87,88,89,90,91,92,93,94,95,96,97,98,99,100,101,102].

## 2. Sample Pretreatment Methods

Sample extraction and purification is vitally important. In the pretreatment processes of vitamins, different substances can be separated and preconcentrated which can improve the analytical performance significantly (e.g., selectivity, sensitivity, accuracy) [30,31]. In the past, soxhelt extraction and heating under reflux have been the most commonly used methods; both these two methods have limitations due to the high costs of time and organic solvents. With the development of analysis technics, many eco-friendly and effective sample preparation technologies have emerged and are becoming more and more popular. These new sample preparation technologies can significantly decrease the personnel costs and time consumed [49,51,58,83,100,102].

The applied pretreatment methods heavily depend on the type of matrix used. A sample type can be divided into two categories based on its state during the determination of vitamins. Liquid samples and solid samples are most common for liquid samples such as serum, juice, milk and so on. There is no need for grinding, and homogeneity, sonicating and heating reflux are often employed for extraction. Then, LLE is frequently used. For solid samples, such as capsules, tablets, meat and so on, grinding and homogeneity are necessary. After the targets have been extracted, different pretreatment methods are needed. In brief, for different samples, different pretreatment methods have been established, e.g., for most solid samples, homogenization, drying and sieving through a screen were adopted, while for serum, the samples can be processed with deproteinization only.

### 2.1. Protein Precipitation, Centrifugation and Filtration

Samples such as whole blood, serum, plasma or urine are a complex mixture of biologically active compounds that can bond to the target analyte, interfere with determination or have a negative impact on the stability of the observed compound. To increase compatibility with the detection and separation techniques, some sample preparation procedures are worth considering, such as protein precipitation, centrifugation and filtration [36,38,40,42,45,48,55,59].

Modern detection tools are far more sensitive and some samples are relatively simple to collect and require only a few steps to purify. Methodology using a “dilute and shoot” technique is gaining popularity and is now frequently used. Ciulu et al. [36] published a method for honey samples that used a diluted mixture of 1 mL of 2 M NaOH with 12.5 mL of 1 M phosphate buffer followed by filtration through a PVDF membrane filter. Dabre et al. [42] published a method for vitamin tablets that used a solved and diluted solution followed by filtration through an 0.2 μm PTFE membrane filter. Leacock et al. [45] developed a method for energy drinks that used a diluted mixture of 60:40 phosphate buffer/methanol solution and then delivered the sample to the detecting instrument. Different diluents were used for different targets; among the diluents, phosphate buffer was the most commonly used diluent [36,45]. Buffer can reduce the fluctuation of pH effectively and reduce the effect of pH on retention time.

Gershkovich et al. [38] described a protein precipitation method for rat plasma centrifuged at 10,000 rpm for 10 min at 5 °C followed by the addition of ice cold acetonitrile. After being filtered using PVDF 0.22 mm centrifugal filters, the supernatant was transferred into autosampler vials and directly measured by HPLC. Brian et al. [40] described a protein precipitation method for plasma that had been centrifuged after the addition of TCA using a 96-well protein precipitation plate filtration system.

Considering that some precipitation reagents used were not directly compatible with the chromatographic equipment, a complete set of protein precipitation reagents were proposed and documented. Still, the most abundantly used reagents include methanol, acetonitrile and a mixture of deionized water with different concentrations of acids.

### 2.2. Ultrasonic Assisted Extraction

**Figure 1:**
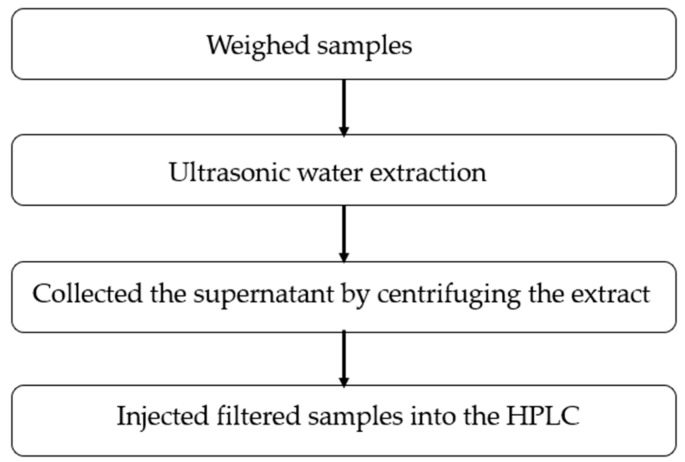
Information block diagram of UAE.

As a high efficiency pretreatment method, UAE can save time and increase the yield and the quality of an extract dramatically [101]. The extraction efficiency can be enhanced by ultrasonic energy through induced cavitation. Since 2010, UAE has gained popularity and is now frequently used [34,42,43,47,52,66,69,76,77,78,79,101]. The information block diagram of UAE is shown in Figure 1.

There are many commercial instruments to choose; thus, the UAE instruments are easy to use. After ultrasonic, we can collect the solvents by filtration or centrifugation easily. In general, after extraction, centrifugation or filtration is inevitable. Chen et al. [101] investigated the effects of different factors on the extraction efficiency of vitamins B from rice bran powder. Different factors (e.g., the extraction time, the ratio of the solvent to solid ratio) were optimized by a two-factor center composite response surface method. The extraction time affects the efficiency of extraction significantly. When the solvent to solid ratio is fixed, the increase in extraction time can significantly improve the vitamin B content in the defatted rice bran extract. Good purification (purification factor is 4.55) and recovery (recovery rate is 92.8%) can be obtained using at 323 K at a solvent to solid ratio of 10.0 for 1.5 h in dried, defatted rice bran extract. Unlike most drugs, most vitamins are sensitive to oxidation and ultraviolet light; thus, some antioxidant measures are often carried out. Francisco et al. [35] established an HPLC method for the analysis of vitamins D and E in milk, fruit juice and a vegetable beverage. During the extraction process, saponification was adopted in a nitrogen atmosphere and darkness. Further, the addition of ascorbic acid or BHT as an antioxidant was employed. Muhammad et al. [41] optimized and validated a liquid chromatography (LC) method for fat-soluble vitamins in human serum. Ascorbic acid and BHT were used as stabilizers in order to prevent oxidation during processing and storage.

Compared with the other pretreatment methods (e.g., heating reflux), the UAE methods mentioned above have shorter extraction times with good recovery. However, the methods still consume large volume of solvents.

### 2.3. Liquid–Liquid Extraction

Another complementary method for the extraction and purification of vitamins is LLE [33,35,41,44,54,57,61,62,63,64,67,68,72,73,75,80]. Although it is now experiencing a renaissance, the use of highly toxic volatile compounds makes this procedure experimental and not suitable for clinical and routine use for high throughput analysis.

Fariborz et al. [44] described an LC method for fat-soluble vitamins using the LLE process. Multivitamin syrup was transferred to a test tube, and then ascorbic acid, n-hexane–DEE solution (9:1, *v*/*v*) and DMSO were added. After that, vortex and centrifugation were carried out, and then the upper organic layer was gathered. After undergoing extraction four times, the organic phases were joined, evaporated to dryness and the residue was redissolved before determination. This can be taken as the normal procedure of LLE. Francisco et al. [35] compared the extraction efficiencies of different regents. Extraction with diethyl ether and hexane (2 × 50 and 1 × 100 mL) was tested, and the best results were obtained using hexane for the extraction of vitamins D and E. Muhammad et al. [41] optimized and validated an LC method for several fat-soluble vitamins in human serum. During the extraction process, n-hexane, chloroform, diethyl ether, ethyl acetate, and dichloromethane were compared in terms of their extraction efficiency on the targets. The best recovery of the targets was obtained using a two-step extraction process (n-hexane followed by dichloromethane). The above pretreatment methods are mainly used to purify fat-soluble vitamins. For water-soluble vitamins, LLE methods have also been developed. Cloud-point extraction is an alternative method. Berton et al. [57] developed an LC method for vitamin B_12_ using the ionic liquid-based aqueous two-phase system extraction process. The urine sample was transferred to a vial after being centrifuged, and then the ionic liquid (0.2 g of [C_6_ mim] [Cl]) was added and fully dissolved into the pretreated sample. Then, K_2_HPO_4_ (3 g) was added after being vortexed, the homogeneous solution became cloudy and VB_12_ was extracted into the ionic liquid (IL) phase. After five min of stirring (without vortex assistance), two well-defined phases were formed. VB_12_ was gathered in the upper IL-enriched phase which can be directly injected in to the detecting instruments. During the pretreatment process, variables such as pH, temperature and the composition of aqueous two-phase system (ATPS), which can affect the IL-based ATPS approach, were optimized. The average extraction efficiency was over 95% under optimum conditions.

### 2.4. Dispersive Liquid–Liquid Micro-Extraction

Different miniaturized pre-treatment techniques based on LLE were developed prior to 2010, including SDME, HF-LPME and so on. Since 2010, DLLME has become a very popular environmentally benign sample preparation technique, because it has a lot of advantages, such as low solvent cost and high enrichment factor [60,85].

In the work of Viñas et al. [60], DLLME with HPLC-PDA detection and a comparison with MS/MS detection for vitamins D and K in foods were combined. For the DLLME procedure, the targets were extracted with acetonitrile (3 mL) which was also used as dispersive solvent. Then, an extractant solvent (carbon tetrachloride, 150 μL) was added, the mixture was injected into water directly using a micropipette, and after being shaken and centrifuged, the demented phase was collected and evaporated to dryness. The residue was reconstituted and injected into the LC. This method eliminates interfering compounds in the matrix, is sensitive and has an improved limit of detection (LOD) compared to other methods. Zeeb et al. [85] established a simple and accurate technique for the determination of vitamin B_1_ using the DLLME procedure to purify interfering substance in tablets and urine samples. Under optimum conditions for DLLME, 10.0 mL of sample solution containing the analytes (pH = 13) was transferred into a glass test tube with a conic bottom. One milliliter of ferricyanide solution (0.01 mol·L^−1^) was added to sample and mixed completely. Then, 0.50 mL of acetone (disperser solvent) containing 122 μL of chloroform (micro-extraction solvent) was injected rapidly into the sample solution. Then, the cloudy solution was formed. After that, the dispersed fine droplets of chloroform were sedimented, and the sedimented phase was removed and injected into the analyzer. Subsequently, the samples were determined by spectrofluorimeter. The results showed that this method has a good recovery and limit of quantitation.

Compared with traditional LLE methods, the abundant contact surface of fine droplets and analytes speeds up the mass transferring processes of analytes from the aquatic phase to the organic phase in a DLLME process, which not only greatly enhances the extraction efficiency but also overcomes the time-consumption problem [56,57]. However, the recoveries obtained by the DLLME method are usually not high enough compared with those of other methods. This may be caused by the use of a dispersive solvent which usually decreases the partition coefficients of analytes into the extraction solvents [58,59].

### 2.5. Solid Phase Extraction

SPE is one of the most common methods to pretreat samples and has been applied to analyze vitamins in different matrices [39,49,51,58,71,83,102]. For liquid samples, SPE is generally directly used to treat real samples [83,102]. However, for solid samples, the analytes are extracted from the sample matrices using organic solvents, like acetonitrile, in advance, and then the SPE procedure is performed to the extract [39,49,51,58]. Generally, SPE cartridge columns are activated before successive washing with different agents during the SPE process. Then, the samples are passed through the cartridges at settled flow rates. The cartridges are then dried, and analytes are eluted from the cartridges.

In the SPE process, solid phase materials, which are useful for extraction, concentration and clean-up, are available in a wide variety of chemistries, adsorbents and sizes. The sorbent selected in SPE controls analytical parameters such as selectivity, affinity and capacity. For this reason, different SPE materials have been used. Because of the different chemical properties of water-soluble and fat-soluble vitamins, SPE methods using different columns have been established.

An LC method was proposed to measure water-soluble vitamin B and C using SPE pretreatment methods by Rudenko et al. [39]. The best results in terms of purification efficiency and recovery rate were obtained with the reversed-phase adsorbent Sep-Pak C_18_ column. The adsorbent was preliminary washed with methanol (5 mL) and distilled water (5 mL). Then, 3 mL of the extract of combined feed was passed through the column. After that, the adsorbent was washed with 1 mL of distilled water. Vitamins were desorbed with 3 mL of methanol. The eluate obtained was analyzed after several treatments. Guggisberg et al. [49] established a purification method for vitamin B_12_ in meat samples using an immunoaffinity column. Fifteen millilitres of the supernatant of the homogenized sample was filtered and loaded onto an immunoaffinity column. Ten millilitres of purified water was used to remove impurities, and then 3 mL of methanol was used to elute the targets by complete denaturation of the antibody. The eluate was concentrated to dryness and reconstituted in the mobile phase before analysis. The recoveries and limits of detection obtained were satisfactory.

Irakli et al. [58] developed and validated a HPLC method for the simultaneous determination of vitamin E and carotenoids in cereals after SPE. As we know, vitamin E is a kind of fat-soluble vitamin; thus, in addition to the SPE columns mentioned above, new columns were adopted to purify the targets. In the experiment, three columns were compared in regard to their purification effects. OASIS cartridges with CH_2_Cl_2_ as the elution solvent were selected for the extraction of studied targets from cereal samples due to having better recoveries than others. Prior to the extraction of SPE, cartridges were conditioned with 3 mL of methanol and 3 mL of water. Subsequently, the above extracts were applied after the addition of 2 mL water to decrease the percentage of ethanol content in the supernatants and allowed to pass through the bed without suction. After washing with 2 mL of water, the retained constituents were eluted with 2 mL dichloromethane, followed by evaporation to dryness. The residue was reconstituted with 200 μL of methanolic solution of a-tocopherol acetate (IS, 50 μg/mL), and aliquots of 20 μL were injected into an HPLC column. Higashi et al. [71] also established a specific LC-MS/MS method for the determination of vitamin D. During the pretreatment process, the methanolic extracts were combined, diluted with water (400 mL) and purified using an Oasis HLBs cartridge. After successive washing with water (1 mL) and methanol–water (7:3, *v*/*v*, 1 mL), the vitamin D metabolites and internal standard (IS) were eluted with ethyl acetate (500 mL). This method was reproducible (intra- and inter-assay relative standard deviations (RSDs), <6.9%) and accurate (analytical recovery, 95.2–102.7%), and the limit of quantification (LOQ) was 3.0 ng/mL. The developed method enabled specific quantification of vitamin D and its metabolites.

SPE methods have shown good behavior in the process of purification producing the desired clean-up effect and achieving automation. With more and more sorbents been developed, more and more SPE columns are becoming optional. Liu et al. [102] developed a novel packed-fiber solid phase extraction procedure based on electrospun nanofibers in human serum. The parameters affecting extraction efficiency were optimized. The LOD for retinol was 0.01 μg/mL and it was 0.3 μg/mL for α-tocopherol. The relative recovery was >90%, which meets the requirements of the analyses of retinol and α-tocopherol in human plasma with satisfactory results.

SPE has many advantages compared with other extraction methods, e.g., complete phase separation, high recovery and low consumption of organic solvents. However, during the process, breakthrough problems may occur. With the development of pretreatment technology, online SPE has been developed to purify some targets. We researched the literature published after 2010, but found no papers that used automatic sample pretreatment techniques to purify vitamins.

### 2.6. Supercritical Fluid Extraction

During the process of SFE, supercritical fluids (usually CO_2_) are adopted as extraction media. Supercritical CO_2_ has the advantages of high diffusivity and low viscosity which helps it to diffuse through solid materials easily. The characteristics of supercritical fluids allow faster extraction compared with traditional LC. Chen et al. [101] studied the recovery of B vitamins from rice bran powder. Before the UAE process, the rice bran was degreased using carbon dioxide and the degreasing effect was good.

### 2.7. Brief Summary

Among all the sample preparation methods, reflux, UAE and SFE are preferred for solid samples, while for liquid samples, LLE, SPE and DLLME are preferred. Reflux extraction methods are traditional methods involving the consumption of large amounts of organic solvents and extraction time. A high extraction efficiency can be obtained with SFE, but expensive instruments are required compared with UAE. Considering the column passing operation, methods like SPE can be complicated. However, multiple samples can be prepared simultaneously by SPE; thus, the total time required can be greatly saved. Moreover, for SPE, it can be coupled with LC to achieve online analysis.

In the study of sample preparation methods, parameter optimization is very important. If the optimization of a method is very complete, it is still a drawback. To find the conditions to allow fast and efficient extraction or clean-up of the target compounds from the sample matrix, the fractional factorial design has been used in some studies to investigate the influences of the extraction conditions [101]. Moreover, the selected design allows the interpretation of results using statistical tests and graphic tools to determine which factors have statistically significant effects as well as to determine which interactions are significant between factors.

## 3. Analysis Methods

Analytical methods are often divided into three groups—screening, quantitative and confirmatory—according to different purposes. Screening methods are specifically designed to avoid untrue results. They usually offer semi-quantitative results and determine analytes at the level of interest. Quantitative methods are often used for the quantification of targets based on different detectors. The ability to reliably identify a compound is the most important feature of confirmatory methods.

The data from the determination methods of vitamins in foods [34,36,39,43,45,46,48,49,50,53,58,59,60,61,63,65,66,67,68,69,70,74,75,76,77,86,87,89,90,93,94,95,96,97,100,101], drugs [35,37,39,42,44,46,51,52,56,78,79,80,82,84,85,88,92] and biological fluids [33,38,39,40,41,47,55,57,62,64,71,72,73,91,99,102] using chromatography methods [33,34,35,36,37,38,39,40,41,42,43,44,45,46,47,48,49,50,51,52,53,54,55,56,57,58,59,60,61,62,63,64,65,66,67,68,69,70,71,72,73,74,75,76], immunochemical methods [99], capillary electrophoresis [77,78,79,80,81] and so on [82,83,84,85,86,87,88,89,90,91,92,93,94,95,96,97,98,99,100,101,102] have been summarized. Since 2010, HPLC has become the most common method for the determination of vitamins. Considering the rapid development of HPLC and MS detectors, this technology will play an even greater role.

### 3.1. Liquid Chromatography

Due to its high sensitivity and broad linear range, HPLC has been widely used. At present, RP-HPLC are the most widely used analytical methods for vitamins. With the development of new HPLC equipment, new systems that can tolerate ultra-high pressures have been developed. The new system, which is called ultra-high performance liquid chromatography (UHPLC), uses sub-2-μm-particle columns and improves chromatographic performance significantly, for example, in terms of sensitivity, speed and resolution.

#### 3.1.1. LC Coupled with MS and Multiclass Analyses

Table 4 represents a selection of analytical methods used for the detection of vitamins by RP-HPLC and UHPLC with MS detection reported since 2010.

Among these methods, HPLC-MS/MS, which can also be considered to be a confirmatory method, has become the main analytical technique used for the identification of vitamins due to its higher selectivity and sensitivity than other instrumental methods. Being a confirmatory method, MS detection is used to identify and quantify a substance and can be used to confirm a compound’s molecular structure. The basic principle of this detection technique is measurement of the mass-to-charge (*m*/*z*) ratios of ionized molecules. HPLC-MS/MS is often applied using a triple quadrupole analyzer and a selected reaction monitoring mode. This mode allows for the confirmation of the composition of compounds and provides structural information. In MS/MS, the most intensive ionic fragment from a precursor ion is used for quantification. A less sensitive secondary transition is used as the second criterion for confirmation purposes. This mode also improves the precision and sensitivity of the analysis but does not collect the full scan data. This can limit the availability of the full scan data which could otherwise be used to both identify target analytes and detect additional unknown compounds. The choice of one or another MS approach to monitor certain substances and residues in live animals and animal products can be referred to the European Union Commission Decision 2002/657/EC which established performance criteria and other requirements for analytical methods with different types of detection, including MS. The first step in tandem MS detection is the selection of a precursor ion. The HPLC-MS/MS analysis of vitamins is usually performed with an electrospray ionization (ESI) source operated in positive ionization mode. The protonated molecule [M + H]^+^ was chosen as a precursor ion for quantitation in all developed methods.

One of the advantages of MS/MS is the fact that complete HPLC separation of the target analytes is not necessary for selective detection. However, it is always advisable to have good chromatographic separation in order to reduce matrix effects that typically result in the suppression or, less frequently, in the enhancement of analyte signals. Therefore, short HPLC columns are generally used, considerably speeding up the analysis. As indicated in Table 4, C_18_ reversed phase based columns are widely used for HPLC multiresidue analytical methods.

Because MS detection is incompatible with most mobile phases, volatile organic modifiers should be used when HPLC is coupled to MS. Thus, formic and acetic acid or their ammonium salts are added to acetonitrile–water or methanol–water mixtures. The typical concentrations of modifiers range from 2 to 20 mmol/L. It has been observed that the higher concentrations lead to reduced signal intensities.

HPLC-MS/MS methods have been applied to quantify vitamins in different matrices successfully [59,60,61,62,63,64,65,66,67,68,69,70,71,72,73,74,75]. Midttun et al. [64] simultaneously determined three vitamins in a small amount of human plasma. Mass spectrometric parameters were optimized before analysis. The LOD for trans-retinols were 0.10 μM and 3.3 nM for 25-OH D_2_ and 25-OH D_3_, respectively. Thus, the method is able to meet the requirements for determination and can be applied to biological samples. Besides the fat-soluble vitamins, water-soluble vitamins can also be quantified by HPLC-MS/MS and a good level of sensitivity can be obtained. Fenoll et al. [65] established a HPLC-MS/MS method for measuring ascorbic and dehydroascorbic acids in several fruits (e.g., pepper, tomato, orange and lemon). MS/MS transitions of *m*/*z* 173→143, 71 were used for ascorbic acid (AA) while *m*/*z* 175→115, 87 were used for dehydroascorbic acid (DHAA). The negative ion mode of ESI was chosen. The method was successfully applied for the determination of AA and DHAA without derivatization or oxidation/reduction processes. The major advantages of the method include its simplicity (little sample preparation), speed (analysis time is no more than 5 min) and great sensitivity (LODs were 13 ng/mL for AA and 11 ng/mL for DHAA, respectively). The advanced instrument can also be used for the determination of fat- and water-soluble vitamins simultaneously. Santos et al. [66] described a HPLC-DAD-MS/MS method to determine both fat-soluble and water-soluble vitamins in green leafy vegetables simultaneously. The LOD and LOQ were 0.07–170 ng/mL and 0.2–520 ng/mL, respectively.

A defining feature of HPLC-MS/MS methods is the high cost of the equipment and the large consumption of organic reagents. With the invention of UHPLC in 2004, UHPLC-MS/MS has had a wide range of applications in recent years. Stevens [63] determined the quantities of vitamin D_2_ and D_3_ in infant formula and adult nutritionals using a HSS C_18_ column (2.1 × 100 mm, 1.8 μm), and the analysis time was less than 3 min which allowed dramatic administrative time savings. Brouwer et al. [70] used an HSS T_3_ column (2.1 × 150 mm, 1.8 μm) to separate vitamin B groups. With 1.8 μm particles, the analytes can be separated in less than 8 min. In general, with a decrease in the number of particles in the stationary phase, the analysis time reduces significantly.

Another inevitable drawback of HPLC-MS/MS is the occurrence of abundant matrix effects, which compromise the quantitative aspects and selectivity of the methods. Extracts from different matrices usually have high contents of organic components, such as lipids, protein, etc. These interfering compounds compete with the analytes to reach the droplet surface positions which affects the maximum evaporation efficiency and hampers ionization of the analytes. These components also increase the viscosity of the sample and the surface tension of the droplets generated from the ESI source, hindering the evaporation of the analytes. Therefore, before analysis using HPLC-MS/MS, matrix effects should be examined. In order to reduce the effects of the matrix effects, different approaches have been developed. Different factors can affect the matrix effects in theory. Firstly, better separation of the matrix compounds from the analytes can reduce the matrix effects; thus, researchers have compared and optimized the columns in order to achieve better separation. Secondly, dilution of the sample extracts and the use of internal standards or matrix-matched calibration are also frequently used [65]. Thirdly, an internal standard can be applied to the matrix effects due to its nearly identical chemical and physical properties. Brouwer et al. [70] investigated the matrix effect of the method that they established. They found that ion suppression or enhancement occurs, with suppression being most pronounced for 5-MTHF and 5, 10-CH+THF; hence, the isotopically labelled standards compensated the ion suppression or enhancement, rendering the matrix effect for all compounds between 85.4 and 103.9. Docros et al. [72] also studied the matrix effect of the quantitative method that they created. The peak area ratio of vitamin K_1_ to its internal standard in plasma was used to calculate the matrix effect. They found that the matrix has a week ion suppression effect on vitamin K_1_ but they seemed to be dependent on each other.

In recent years, great progress has been made in mass detectors. Different mass detectors have been developed, e.g., hybrid triple quadrupole-linear ion trap (QqLIT) instruments. QqLIT is a powerful technique that is used for large-scale screening of targets in real samples with the advantages of the new equipment, such as high resolution and extract confirmatory results. The method is based on a QqQ with the third quadrupole (Q_3_) which can be used as either a conventional quadrupole mass filter or a linear ion trap that combines the advantages of the classical QqQ scanning functionality and the possibility of additional sensitive ion trap scans to allow structural analysis within the same operating platform. Due to its high ion accumulation capacity, this method has improved the full-spectrum sensitivity and provides very promising modes, such as enhanced full mass scan and enhanced product-ion and multi-stage scans. All of these features make the technique very powerful for the identification of unknown or suspected analytes, even those with poor fragmentation and at low concentrations. Another attractive capability of QqLIT for semi-targeted analysis is its information dependent acquisition that can automatically combine a survey scan with the dependent (enhanced trap) scan during a single experiment. Trenerry et al. [75] described robust methods using HPLC-QqLIT method and measured the serum vitamin D_3_ in different matrices. The level of vitamin D_3_ in fresh bovine milk (0.05 μg/100 mL), commercial (natural and fortified) milk samples (0.01–2 μg/100 mL) and a dairy based infant formula (8 μg/100 mL) was obtained without the need for extensive clean-up procedures. The LOQs were 0.01 μg/100 mL and 0.02 μg/100 mL for LC-MSn and LC-MS/MS, respectively.

#### 3.1.2. Liquid Chromatography Coupled with Other Techniques

Classical reversed-phase HPLC with ultraviolet (UV), photodiode array (PDA) and fluorescence detectors [33,34,35,36,37,38,39,40,41,42,43,44,45,46,47,48,49,50,51,52,53,54,55,56,57,58] is still widely used for the routine quantification of vitamins in different types of samples. All of these approaches are quantitative but not confirmatory, as they cannot provide direct evidence of the structure or composition of a substance. UV detection is the most affordable and versatile method, but the least selective and sensitive, while FL detection is much more sensitive and selective.

The vast majority of chromatographic separations of vitamins have been performed with conventional silica-based reversed phased columns (mainly C_18_) with spherical sorbent particles, 3–5 μm in diameter. The speed of the analysis can be increased through the use of a high temperature or ultra-high pressure system [33,34,35,36,37,38,39,40,41,42,43,44,45,46,47,48,49,50,51,52,53,54,55]. Considering the instability of some vitamins, such as vitamin C, the high column temperature is rarely used. Momenbeik et al. [44] established an HPLC method for the determination of vitamins A, D_3_, E and K. A Zorbax-eclipse XDB-C_8_ column (150 × 4.6 mm, 5 μm) was employed in the experiment, with the wavelength set at 285 nm. This method has been validated and found to be applicable for routine analyses, but the analysis is too long—30 min. Lorencio et al. [33] assessed the suitability of UHPLC for the simultaneous determination of vitamins A, E and D. The HSS T3 cloumn (2.1 × 100 mm, 1.8 μm) was employed. With the decrease in particles in the stationary phases, the analysis time was much shorter. The method consumes no more than 4 min which improves the efficiency significantly. Klimcazk et al. [55] made a comparison of the UPLC and HPLC methods for the determination of vitamin C. The two methods are both applicable for the determination of vitamin C in routine analyses, while UPLC is faster, more sensitive and more environmentally friendly.

Methanol–water and acetonitrile–water are the most common mobile phases. In addition, three-component mixtures have been reported researchers: water, methanol and acetonitrile [58,72]. In most cases, the mobile phase is modified with acetic, formic acid, acetate acid and so on. In addition, the application of an elution gradient is often adopted.

A simple and rapid method for the simultaneous determination of seven water-soluble vitamins in infant milk and dietary supplement uses LC coupled to a corona-charged aerosol detector. The detection limits range from 0.17 to 0.62 mg/L for dietary supplements and 1.7 to 6.5 mg/L for infant milk. The method is more sensitive than the common UV or DAD methods.

#### 3.1.3. Summary

Multiclass analytical methods have been developed due to progress in chromatography and mass spectrometry methods. This has led to significant trends in the detection of different vitamins in complex samples. The progress in chromatography and mass-spectrometry methods has resulted in the development of multiclass analytical methods which are currently a significant trend in the detection of different vitamins in food and biological samples that can be successfully applied for both quantification and screening purposes. These methods are able to detect both fat- and water-soluble compounds and are of great interest to analytical laboratories due to their simplicity, high sample throughput and cost-effectiveness.

### 3.2. Electrophoretic Methods

Capillary electrophoresis (CE) is another good quantitative analytical approach that is mainly used when only small amounts of a sample are available. It is a highly efficient, fast and lower solvent-consuming technique in which sample components are separated according to their sizes and charges. Some advantages of CE are its high separation efficiency, ability to analyze several samples simultaneously in multicapillary systems and low consumption of reagents and accessories (packaged columns are not required). Several CE methods have been published and reviewed for the analysis of vitamins since 2010 [77,78,79,80].

For the past 6 years, CE has been used for the determination of vitamins in different samples [77,78,79,80]. Phosphate and borate buffers which sometimes contain additional organic modifiers, such as sodium polystyrene sulfonate, have been used as running buffers.

Micellar electrokinetic chromatography (MEKC) is the most popular mode of CE. It allows the separation of both ionic and neutral analytes. Applications of the MEKC method as determination methods for the analysis of vitamins have been reported [77,78,79,80]. In the work of Danielle et al. [77], to optimize the electrophoretic method, the composition and concentration of the buffer solution, SDS and organic modifier (ethanol) concentrations, pH, temperature, injection time, injection pressure and the inner diameter of the capillary were evaluated. In this work, a good separation of ten water-soluble vitamins was obtained in only 18 min. This analytical procedure is precise (RSD < 6%), accurate (better than 9%), selective, sensitive, robust and simple. Yin et al. [78] examined different conditions, such as microemulsion composition (effect of surfactant, co-surfactants, oil phases, organic solvents, pH and concentration of the buffer.) and the effects of voltage and temperature. After being optimized, a novel microemulsion system consisting of 1.2% (*w*/*w*) sodium lauryl sulphate (SDS), 21% (*v*/*v*) 1-butanol, 18% (*v*/*v*) acetonitrile, 0.8% (*w*/*w*) *n*-hexane and 20 mM borax buffer (pH 8.7) was applied. Aurora-Prado et al. [79] described a rapid determination method for water- and fat-soluble vitamins simultaneously in commercial formulations by MEKC. The final selected buffer contained SDS (surfactant), butan-1-ol (co-surfactant), ethyl acetate (oil) and pH 9.2 tetraborate buffer, modified with 15% (*v*/*v*) 2-propanol. The UV detection was set at 214 nm which gave adequate sensitivity without interference from sample excipients. Under the optimized conditions, the vitamins were baseline separated in less than 7 min, and acceptable limits of quantification between 8.40 and 16.23 μg/mL were obtained. The method was considered appropriate for rapid and routine analyses.

### 3.3. Microbiological Assays

Based on the fact that specific vitamins are necessary for the growth of specific bacteria, microbiological inhibition assays were established. Because they offer biological responses to vitamin activity and allow the determination of different vitamins, microbiological assays have been recognized by international official institutions as the gold standard for many years. However, the traditional microbiological method still suffers from poor precision and accuracy—a relative measurement uncertainty of ±20% is commonly observed. In the past, this microbiological method has often been used as a screening approach, but with the development of technology in recent years, quantitative methods have been established and the precision and accuracy have improved a lot.

Zhang et al. [100] established a “three-in-one” pretreatment method to determine several water-soluble vitamins using VitaFast kits. The VitaFast kits are fast and friendly to users. The experimental results of the assays which have employed “three-in-one” sample preparation methods are in good agreement with those obtained from conventional VitaFast extraction methods. The proposed new sample preparation method will significantly improve the efficiency of infant formulae inspection.

### 3.4. Biosensors

Since 2010, it has been demonstrated that optical biosensors are excellent tools for detecting vitamins in different matrices [89,90,91,92,93,94,95,96,97]. Their main advantages are their technical simplicity, low cost and the possibility of being used in field analyses.

Electrochemical sensors based on the use of receptors fabricated through different imprinting approaches have been developed for the detection of vitamins [89,90,91,92,93,94,95,96,97]. Trace quantities of vitamins B_1_, B_2_ and C were successfully detected in a microfluidic device by employing electrokinetic separation and electrochemical detection using silver liquid amalgam film–modified silver solid amalgam annular band electrodes (AgLAF–AgSAE). The method is based on the adsorptive accumulation of analytes at the AgLAF–AgSAE in a phosphate buffer (VB_1_), a phosphate buffer with Triton X-100 (VB_2_) and an alkaline borate buffer with Triton X-100 (VC). The analytical parameters and procedure of electrode activation were optimized. The calibration graphs obtained for vitamins C, B_1_ and B_2_ were linear, respectively, for the concentration ranges 0.05–12, 0.01–0.1 and 0.05–3 mg/L. The detection limits were calculated and equaled 0.02, 0.003 and 0.009 mg/L, while the repeatability of the peak current was 2%, 1% and 3%, respectively [92]. On the basis of conventional electrodes, modified electrodes were developed. Revin et al. [91] simultaneously determined several vitamins using a heterocyclic conducting polymer modified electrode. The research compared a bare GC electrode and the proposed 3-amino-5-mercapto-1,2,4-triazole modified glassy carbon (p-AMTa) electrode. The former failed to show stable voltammetric signals for the targets while the p-AMTa electrode showed stable voltammetric signals for the vitamins in a mixture with potential differences of 670 mV and 530 mV between riboflavin (RB)-ascorbic acid (AA) and AA-folic acid (FA), respectively. Nie et al. [93] used electroactive species-doped poly(3,4-ethylenedioxythiophene) films to enhance the sensitivity for the electrochemical simultaneous detection of vitamins B_2_, B_6_ and C. The functionalized PEDOT films were prepared by incorporation of two electroactive species: ferrocene carboxylic acid (Fc-) and ferricyanide (Fe(CN)64-). After comparison, the authors reported that the oxidation peak currents of vitamins obtained at the glassy carbon electrodes (GCEs) modified with electroactive species-doped PEDOT films were much higher than those at the ClO_4_-doped PEDOT films and bare GCEs.

Pisoschi et al. [89] developed a determination method for vitamin C in fruit samples by differential pulse voltammetry. Four hundred and seventy millivolts on the carbon paste working electrode and 530 mV on the Pt strip working electrode were used for the determination of ascorbic acid. The influence of the operational parameters on the analytical signal was investigated. The obtained calibration graph showed a linear dependence between the peak height and the ascorbic acid concentration within the range of 0.31–20 mM with a Pt working electrode and within the range 0.07–20 mM with a carbon paste working electrode. The developed method was applied for the determination of vitamin C in wine and juice samples. A quantity of 6.83 mg/100 mL vitamin C was detected using this method.

One of the prospective trends in this field is the development of nanoparticles, quantum dots and nanocomposites. For example, a facile one-pot strategy for the electrochemical synthesis of poly (3,4-ethylenedioxythiophene)/Zirconia nanocomposite has been applied to analyze vitamins B_2_, B_6_ and C [97]. The obtained PEDOT/ZrO_2_NPs nanocomposite film showed a large specific area, high conductivity, rapid redox properties and the presence of encapsulated structures, which make it an excellent sensing platform for sensitive determination of the targets. Detection limits of 0.012 μM, 0.20 μM and 0.45 μM were obtained for vitamin B_2_ (VB_2_), vitamin B_6_ (VB_6_) and vitamin C (VC), respectively. Jamali et al. [94] described a novel nanosensor based on Pt:Co nanoalloy ionic liquid carbon paste electrode for the voltammetric determination of vitamin B_9_ in food samples. The sensor exhibited an enhanced effectiveness for the electro-oxidation of vitamin B_9_ in aqueous solution. The detection limit was 4 × 10^−8^ M. The proposed modified electrode has several advantages, such as being simple, having high stability, high sensitivity, and excellent catalytic activity, long-term stability and remarkable voltammetric reproducibility for the eletro-oxidation of vitamin B_9_. Baghizadeh et al. [90] determined a voltammetric sensor for simultaneous determination of vitamin C and B_6_ in food samples using a ZrO_2_ nanoparticle/ionic liquids carbon paste electrode. At an optimum condition (pH 7.0), the two peaks separated into ca. 0.44 and 0.82 V for AA and vitamin B_6_, respectively. The detection limits for AA and vitamin B_6_ were 0.009 and 0.1 μM, respectively. The modified electrode has been successfully applied for assays of AA and vitamin B_6_.

Another interesting approach is the application of new potentiometric sensors with Donnan potential as analytics. The Donnan potential at the interface between the ion-exchange polymer and the solution of an electrolyte represents the difference between the Galvani potentials in the ion–exchanger phase and solution phase. Its value can be estimated if one measures the electromotive force (EMF) of the electrochemical circuit. Bobreshova et al. [95] determined the quantities of amino acids, vitamins and drug substances in aqueous solutions using new potentiometric sensors with Donnan potential. Certain regularities of the Donnan potential formation have been studied in systems with polymers of different structures and solutions containing inorganic ions and organic electrolytes in different ionic forms. The developed sensor was introduced as a cross-sensitive electrode into the array of multisensor systems for multicomponent quantitative analysis and the measurement error of electrolytes in aqueous solutions did not exceed 10%.

### 3.5. Spectrometry

Fluorescence spectrometry can be used as a screening method to detect vitamins in different samples [84,85]. In a previous paper [85], DLLME combined with spectrofluorimetry was applied to the extraction, pre-concentration and analysis of thiamine (vitamin B_1_). The method proposed to detect vitamins was based on the oxidation of thiamine with ferricyanide to form fluorescent thiochrome (TC). The excitation wavelengths and emission wavelengths were 375 and 438 nm, respectively. After the optimization, the detection limit reached 0.06 ng/mL. The method was successfully applied to pharmaceutical formulations and human urine. Mallboud et al. [84] described two spectrofluorimetric methods for the detection of some water-soluble vitamins. The first proposed method depends on the oxidation of thiamine to fluorescent thiochrome using iodine/NaOH, while the second method depends on using an acetate buffer of pH 6 for the simultaneous determination of riboflavin and pyridoxine HCl using their native fluorescence levels. Different variables that affect the fluorescence intensity related to the two methods were optimized. The excitation wavelengths and emission wavelengths of thiochrome were 375 and 438 nm, while pyridoxine and riboflavin exhibited intrinsic native fluorescence with excitation and emission maxima at 325, 457 and 415, 527 nm, respectively. Good recoveries and sensitivities were obtained, and the proposed methods were applied to the analysis of the investigated vitamins in their laboratory-prepared mixtures and pharmaceutical dosage forms.

A simple, sensitive and accurate UV derivative spectrophotometric method for the detection of caffeine and vitamin B groups in energy drinks has been developed [83]. Caffeine was determined in a mixture with vitamin B_2_ with the zero-crossing technique from the I derivative spectra (λ = 266.8 nm), and vitamin B_3_ in mixture with vitamin B_6_ vitamin from the II derivative spectra (λ = 280.1 nm). Vitamin B_12_ was also been determined in a three-component mixture with vitamins B_3_ and B_6_. Mohamed et al. [88] also described the use of two spectrophotometric methods—derivative and multivariate methods—for the determination of several water-soluble vitamins, the described methods were successfully applied for the determination of vitamin combinations in synthetic mixtures and dosage forms from different manufacturers. The methods are sensitive enough for routine analysis. Monakhova et al. [87] established a chemometrics-assisted spectrophotometric method for the simultaneous detection of vitamins in complex mixtures. The main contribution of the method was the advanced independent component analysis algorithm. The key features of the proposed method are its simplicity, accuracy and reliability. Through comparison with some other established methods (MCR-ALS, SIMPLISMA, other ICA techniques), the proposed method was shown to be comparable or even outperformed other chemometrics methods.

Near-infrared spectroscopy (NIR) methods have been applied for the determination of the vitamin B group [86]. The NIR spectra of the samples were acquired from 1350 nm to 1800 nm. The multivariate regression models obtained by NIR spectroscopy and the PLS method showed a low rate of predictive errors and good correlation coefficients.

### 3.6. Other Methods

Besides the methods mentioned above, several effective methods have also been developed, e.g., immunoassays [99], high performance thin-layer chromatography [81] and supercritical fluid chromatography [98]. The use of these methods is justified when it is necessary to carry out routine quality control of relatively simple sample compositions. The advantages of these methods include their simplicity, compactness and relatively low cost of the analysis.

Immunoassays are characterised by their high specificity, high sensitivity, simplicity and cost effectiveness which makes them particularly useful for routine uses. These assays are based on a specific reaction between an antibody and an antigen, and they are capable of detecting the low concentration of residues in a short period of time and often do not require laborious extraction or clean-up steps. Enzyme-linked immunosorbent assays (ELISA) are the most widely used immunoassays due to their high sample throughput. These methods can drastically reduce the number of analyses required to detect vitamins in different samples. Martin et al. [99] used the 25-OH vitamin D ELISA Assay kit to determine the quantity of vitamin D; the method was successfully applied into serum samples.

Recently, TLC has been improved to incorporate HPTLC grade stationary phases, automated sample application devices, a controlled development environment, automated development, forced-flow techniques, computer-controlled densitometry, quantitation and fully validated procedures. HPTLC is becoming a routine analytical technique because of its advantages of low operating cost, high sample throughput, simplicity, speed, need for minimum sample clean up, high reproducibility, accuracy, reliability and robustness [81]. Panahi et al. [81] isolated and quantified vitamins B_1_, B_2_, B_6_ and B_12_ using HPTLC. The precoated aluminum-backed silica gel G60 F254 HPTLC plate was developed with nearly 30 different solvent systems. The ethanol-chloroform–acetonitrile–toluene–ammonia–water (7:4:4.5:0.5:1:1) mixture was selected as the mobile phase, and the wavelength was set at 254 nm. The retention factors of vitamins B_1_, B_2_, B_6_ and B_12_ were 0.36, 0.6, 0.85 and 0.46, respectively, the LOQ were 141.72, 42.41, 100.31 and 11.5 ng and the LOD were 42.52, 12.72, 30.09 and 3.45 ng, which is applicable for routine analysis.

SFC is a complementary separation technique to GC and LC. This technique employs supercritical carbon dioxide or subcritical carbon dioxide as the mobile phase. As mentioned above, SFC could be applied into purify the vitamins in rice [101], SFC could also be used to separate the vitamins. Taguchi et al. [98] simultaneously separated and quantified water- and fat-soluble vitamins using a single chromatography technique to unify SFC and LC. In this method, the phase state was continuously changed in the following order: supercritical, subcritical and liquid. The gradient of the mobile phase starting at almost 100% CO_2_ was replaced with 100% methanol at the end. As a result, this approach achieved further extension of the polarity range of the mobile phase in a single run and successfully enabled the simultaneous analysis of fat- and water-soluble vitamins with a wide log P range of −2.11 to 10.12. Furthermore, the seventeen vitamins were exceptionally separated in 4 min. The results indicated that the use of dense CO_2_ and the replacement of CO_2_ by methanol are practical approaches in unified chromatography covering diverse compounds. In conclusion, the SFE method has significant advantages, including (1) the method is environmentally friendly, because the CO_2_ is non-flammable and has no negative impact on human health; (2) The super fluid is flexible in adjusting its dissolving power by adding different co-solvents; (3) SFE can inherently eliminate organic solvents and provide cleaner extracts at the same time. The only serious drawback of SFE is its higher investment costs compared to traditional atmospheric pressure extraction techniques.

### 3.7. Summary of Analysis Methods

Among all the analytical methods, LC is the most popular due to its advantages. LC methods can meet the requirements of the qualitative and quantitative analysis of vitamins in different matrices, such as foods and biological samples, especially when it is combined with MS. However, UV and FLD detectors suffer overlapping peaks when dealing with complex samples, while matrix effects and high costs are necessary when using LC-MS. CE is alternative method for the determination of vitamins which is highly efficient, low solvent-consuming and fast, but its separation reproducibility needs to be enhanced. The main advantages of biosensors include their low cost, technical simplicity and the possibility of being used in field analyses, while their relatively short lifespan restricts the development of technology. Spectrometry is cheap and easy to promote, but its sensitivity does not quite meet requirements sometimes. In a word, with the development of equipment of HPLC and MS, this technology will surely be broadly used, while other technologies, such as electrophoresis and spectrometry, are seen as supplementary methods to be used when necessary.

## 4. Conclusions

Since 2010, different methods for determination of vitamins in various types of samples have been proposed. The key roles of these methods are played by sample preparation techniques, and the main efforts in this field have been focused on the optimization of the preparation, extraction and clean-up steps and on the enhancement of the environmental safety of these procedures. The method with the most promise in achieving these goals is SPE. The main advantages of this approach are its good compatibility with high throughput multiresidue analytical procedures and its relatively low cost. Therefore, this technique is expected to have the most pronounced development in the future.

The currently proposed analytical approaches for the detection of vitamins are mainly based on HPLC–MS or HPLC-MS/MS. Great advances in HPLC-MS/MS have made it a key technique for the determination of not only vitamins but also other targets. The main trend in this field is the combination of MS detectors with modern chromatographic approaches such as UHPLC and the application of the powerful QqTOF and Orbitrap instruments. These hybrid approaches have made a great contribution to the analysis of trace organic contaminants, including vitamins, and have contributed to the development of multianalyte techniques for the detection of a wide range of substances in a single analytical run. These methods seem poised to be the most frequently used techniques for the purposes of analysis in the future. The main disadvantages of these methods are their complex equipment and high cost. There is currently great interest in the development of screening methods based on microbiological, immunoassays and biosensors, which have the main advantages of being low cost, having short analysis times and the possibility of their onsite use. The clear trend in this field is the miniaturization of screening systems (chips, microarrays, microtiter plates) as well as their automation. We think that these features will maintain the sustainable progress of these methods in the near future.

## Figures and Tables

**Table 1:** List of fat-soluble vitamins [13].

Vitamin Name	Function	Dietary Sources
Vitamin A	Helps with (1) healthy mucous membranes; (2) skin, vision, tooth and bone growth; (3) health of the immune system.	From animal sources (retinol): liver, eggs, fortified margarine, butter, cream, cheese, fortified milk.
		From plant sources (beta-carotene): dark orange vegetables (pumpkin, sweet potatoes, winter squash, carrots), fruits (cantaloupe, apricots), dark green leafy vegetables.
Vitamin K	Required for correct blood clotting.	Vegetables from the cabbage family, leafy green vegetables, milk; it is also produced in the intestinal tract by the bacteria.
Vitamin E	Helps to protect the cell walls.	Nuts and seeds, egg yolks, liver, wholegrain products, wheat germ, leafy green vegetables and polyunsaturated plant oils.
Vitamin D	Required to properly absorb calcium.	Fortified margarine, fortified milk, fatty fish, liver, egg yolks; the skin can also produce vitamin D when it is exposed to sunlight.

**Table 2:** List of water-soluble vitamins [13].

Vitamin Name	Benefits	Dietary Sources
Ascorbic Acid (Vitamin C)	Ascorbic acid is an antioxidant, and it is a portion of an enzyme that is required for protein metabolism. It also helps with iron absorption and is important for the health of the immune system.	Found in vegetables and fruits, especially: kiwifruit, mangoes, papayas, lettuce, potatoes, tomatoes, peppers, strawberries, cantaloupe and so on.
Thiamine (Vitamin B_1_)	Thiamine is a portion of an enzyme that is required for energy metabolism, and it is important for nerve function.	Found in moderate amounts in all nutritious foods: nuts and seeds, legumes, wholegrain/enriched cereals and breads, pork.
Riboflavin (Vitamin B_2_)	Riboflavin is a portion of an enzyme that is required for energy metabolism. It is also important for skin health and normal vision.	Enriched, wholegrain cereals and breads, leafy green vegetables, milk products.
Niacin (Vitamin B_3_)	Niacin is a portion of an enzyme that is required for energy metabolism. It is also important for skin health as well as the digestive and nervous systems.	Peanut butter, vegetables (particularly leafy green vegetables, asparagus and mushrooms), enriched or wholegrain cereals and breads, fish, poultry and meat.
Pantothenic Acid (Vitamin B_5_)	Pantothenic acid is a portion of an enzyme that is required for energy metabolism.	It is widespread in foods.
Pyridoxine (Vitamin B_6_)	Pyridoxine is a portion of an enzyme that is required for protein metabolism. It also helps with the production of red blood cells.	Fruits, vegetables, poultry, fish, meat.
Folic Acid	Folic acid is a portion of an enzyme that is required for creating new cells and DNA.	Liver, orange juice, seeds, legumes, leafy green vegetables. It is now added to many refined grains.
(Vitamin B_9_)		
Cobalamin (Vitamin B_12_)	Cobalamin is a portion of an enzyme required for the production of new cells, and it is important to the function of nerves.	Milk, milk products, eggs, seafood, fish, poultry, meat. It is not present in plant foods.
Biotin (Vitamin H)	Biotin is a portion of any enzyme that is required for energy metabolism.	It is widespread in foods and can be produced by bacteria in the intestinal tract.

**Table 3:** Pretreatment methods, sample matrices and targets of the recent articles.

Pretreatments	Determination Methods	Sample Matrix	Analytes	Ref.
liquid–liquid extraction (LLE)	liquid chromatography-ultraviolet detection (LC-UV)	Human serum	Vitamins A (retinol, retinyl esters), E (α- and γ-tocopherol) and D (25-OH vitamin D)	[33]
ultrasonic assisted extraction (UAE), filtration	LC-UV	Multivitamin capsule	Benfotiamine (B_1_). Pyridoxine hydrochloride (B_6_), mecobalamin (B_12_)	[34]
LLE	LC-UV	Milk, fruit juice and vegetable beverage	Vitamins E (a-, c- and d-tocopherol) and D (cholecalciferol and ergocalciferol)	[35]
Dilute and shoot	LC-UV	Honey	Vitamin B_2_, riboflavin; vitamin B_3_, nicotinic acid; vitamin B_5_, pantothenic acid; vitamin B_9_, folic acid; and vitamin C, ascorbic acid	[36]
UAE, filtration	LC-UV	Mineral tablets	Thiamine, riboflavin, niacinamide, pantothenic acid, pyridoxine, folic acid and ascorbic acid	[37]
Protein precipitation, centrifugation and filtration	LC-UV	Rat plasma	Vitamins D_3_ and K_1_	[38]
supercritical fluid extraction (SPE)	LC-UV	Combined feed, premixes, and biologically active supplements	Ascorbic acid (C), nicotinic acid (B_3_ or PP), nicotinamide (B_3_ or PP), pyridoxine hydrochloride (B_6_), riboflavin (B_2_) and thiamine hydrochloride (B_1_)	[39]
Protein precipitation, filtration	LC-FLD	Plasma	Vitamin B_2_ (riboflavin)	[40]
LLE	LC-UV	Human serum	All-trans-retinol, retinyl acetate, a-tocopherol, a-tocopheryl acetate	[41]
UAE	LC-UV	Vitamin tablets	10 vitamins (7 water-soluble and 3 fat-soluble)	[42]
UAE	LC-UV	Energy drinks	Caffeine and water-soluble vitamins	[43]
LLE	LC-UV	Pharmaceutical formulations	Fat-soluble vitamins	[44]
Dilute and shoot	LC-UV	Red bull and other energy drinks	Caffeine and vitamin B_6_	[45]
UAE	LC-UV	Vitamin premixes, bioactive dietary supplements and pharmaceutical preparations	12 water-soluble vitamins	[46]
UAE	LC-UV	Food samples, human plasma and human adipose tissue	Retinol, tocopherols, coenzyme Q_10_ and carotenoids	[47]
Filtration, dilute and shoot	LC-UV	*A.marmelos* fruit juice	Vitamin C, polyphenols, organic acids and sugars	[48]
SPE	LC-UV	Meat products	Vitamin B_12_	[49]
Extraction, filtration	LC-UV	Leafy vegetables	Riboflavin (vitamin B_2_), niacin (vitamin B_3_), pantothenic acid (vitamin B_5_) and pyridoxine (vitamin B_6_)	[50]
SPE	LC-UV	Leaves of Suaeda vermiculata	Vitamin B group	[51]
UAE	LC-UV	12 multivitamin/multimineral pharmaceuticals and preparations and human serum	B-complex vitamins and vitamin C	[52]
Extraction, filtration	LC-UV	Fruits and vegetables	L-ascorbic and dehydroascorbic acids	[53]
LLE	LC-FLD	Human serum	Vitamin K	[54]
Dilute and shoot	LC-UV	Fruit beverages and in pharmaceutical preparations	Vitamin C	[55]
Dilute and shoot	LC-UV	Pharmaceutical solid dosage	B-group vitamins and atorvastatin	[56]
LLE	LC-UV	Urine	Vitamin B_12_	[57]
SPE	LC-UV	Cereal samples	Tocopherols, tocotrienols and carotenoids	[58]
Extraction, filtration	Flow-Injection MS/MS	Nutritional supplements	B vitamins	[59]
dispersive liquid–liquid microextraction (DLLME)	LC-DAD-MS	Infant foods and several green vegetables	Vitamins D and K	[60]
LLE	LC-MS	Bovine milk	Vitamins A, E and b-carotene	[61]
LLE	LC-MS	Serum	25-Hydroxyvitamin D_3_ and 25-hydroxyvitamin D_2_	[62]
LLE	LC-MS	Infant formula and adult nutritionals	Vitamins D_2_ and D_3_	[63]
LLE	LC-MS	Human plasma	Vitamins A, D and E	[64]
Centrifugation and filtration	LC-MS	Vegetables and fruits	Ascorbic and dehydroascorbic acids	[65]
UAE	LC-DAD-MS	Green leafy vegetables	Fat and water-soluble vitamins	[66]
LLE	LC-MS	Infant formula and adult nutritionals	Vitamins D_2_ and D_3_	[67]
LLE	LC-DAD-MS	Milk	Carotenoids and fat-soluble vitamins	[68]
UAE	LC-MS	Nutritional formulations	Fat- and water-soluble vitamins	[69]
Centrifugation and filtration	LC-MS	Rice	Folates	[70]
SPE	LC-MS	Neonatal dried blood spots	25-Hydroxyvitamin D_3_	[71]
LLE	LC-MS	Blood	Vitamin K_1_	[72]
LLE	LC-MS	Serum	25(OH) Vitamin D_3_ and D_2_	[73]
Centrifugation and filtration	LC-MS	SRM 1849 infant/adult nutritional formula powder	Water-soluble vitamins	[74]
LLE	LC-MS	Milk	Vitamin D_3_	[75]
UAE	LC-corona-charged aerosol detector	Infant milk and dietary supplement	Water-soluble vitamins	[76]
UAE	MEKC	Food supplements	Water-soluble vitamins	[77]
UAE	MEKC	Commercial multivitamin pharmaceutical formulation	Water- and fat-soluble vitamins	[78]
UAE	MEKC	Multivitamin formulation	Water- and fat-soluble vitamins	[79]
LLE	MEKC	Multivitamin tablets and vitamin E soft capsules	Fat-soluble vitamins	[80]
Dilute and shoot	HPTLC	Standard stock solutions	Vitamins B_1_, B_2_, B_6_ and B_12_	[81]
Extraction, dilute and shoot	PCR, PLS and TLC	Pharmaceutical formulations	Vitamins B_1_, B_6_ and B_12_	[82]
SPE	Spectrophotometry	Energy drinks	Caffeine and B vitamins	[83]
Extraction, dilute and shoot	Spectrofluorimetry	Pharmaceuticals	Water-soluble vitamins	[84]
DLLME	Spectrofluorimetry	Tablets and urine samples	Vitamin B_1_	[85]
Filtration	Spectrofluorimetry	Corn steep liquor	Vitamins B_2_, B_3_, B_6_ and B_7_	[86]
Dilute and shoot	Spectrofluorimetry	Multivitamin drugs, food additives and energy drinks	Fat- and water-soluble vitamins	[87]
Dilute and shoot	Spectrofluorimetry	Dosage forms	Water-soluble vitamins	[88]
Centrifugation	Voltammetry	Fruit juices and wine	Ascorbic acid content	[89]
Centrifugation, UAE	Voltammetric Sensor	Food samples	Vitamin C and vitamin B_6_	[90]
Dilution	Electrode	Human plasma	Vitamins B_2_, B_9_ and C	[91]
No previous preparation	Electrode	Pharmaceutical samples and fruit juices	Vitamins C, B_1_ and B_2_	[92]
Dilution	Electrode	Orange juice samples	Vitamins B_2_, B_6_ and C	[93]
Centrifugation, filtration	Nanosensor	Food samples	Vitamins B_9_	[94]
No previous preparation	Sensor	Aqueous solutions	Vitamins B_1_, amino acids and drug substances	[95]
Dilution	Sensors	Polymerization samples	Vitamin B_3_	[96]
Dilution	Nanocomposites	Honey samples	Vitamins B_2_, B_6_ and C	[97]
Dilution	SFC-MS/MS	Standard stock solutions	Water- and fat-soluble vitamins	[98]
Centrifugation	HPLC, ELISA	Serum	Vitamins A, C and D	[99]
SPE	Microbiological assays	Infant formula	B group vitamins	[100]
UAE	LC-UV	Rice bran powder	Vitamins B_1_, B_2_, B_3_, B_6_ and B_12_	[101]
SPE	LC-UV	Plasma	Retinol and α-tocopherol	[102]

**Table 4:** Examples of HPLC-MS/MS methods for the detection of vitamins.

Analysis Time (min)	Instrument Analysis Methods	Column	Mobile Phase	Limit of Detection (LOD)	Limit of Quantification (LOQ)	Ref
45 min	Flow-Injection Tandem Mass Spectrometry (Linear Ion-Trap Mass Spectrometer)	Cadenza CD-C_18_ column (4.6 × 250 mm, 3 μm)	A: 20 mM aqueous ammonium formate (pH 4); B: methanol. Gradient	0.04–48.2 ng/g	0.13–160.6 ng/g	[59]
15 min	HPLC-APCI-MS/MS	Zorbax Eclipse ODS (4.6 × 250 mm, 5 μm)	Acetonitrile, isopropanol and water. Gradient	0.2–0.6 ng/mL	0.8–2 ng/mL	[60]
26 min	High Performance Liquid Chromatography–Ion Trap Mass Spectrometry (HPLC–Msn)	Polaris C_18_ column (2.1 × 150 mm, 5 μm)	A: water; B: methanol. Gradient	no report	0.1 μg/100 mL for all trans-retinol and α-tocopherol and 1 μg/100 mL for β-carotene	[61]
15 min	LC-MS/MS	Zorbax SB-CN column (4.6 × 250 mm, 5 μm)	34% water and 66% methanol. Isocratic	∼0.15 ng/g	no report	[62]
3 min	Ultra-Pressure Liquid Chromatography with Tandem Mass Spectrometry Detection (UPLC-MS/MS)	UPLC HSS C_18_ column (2.1 × 100 mm, 1.8 μm)	A: 2 mM NH_4_COOH; B: 2 mM NH_4_COOH: MeOH. Gradient	The LODs for vitamin D_2_ were reported as 0.20 and 0.61 μg/100 g,	The reported LOQ values for vitamin D_3_ were 0.47 and 1.44 μg/100 g	[63]
6 min	Liquid Chromatography/Tandem Mass Spectrometry	Ascentis Express C_18_ column (4.6 × 50 mm, 2.7 μm)	A: Ammonium formate in MeOH; B: H_2_O. Gradient	0.1 μM for all-trans retinol, 3.3 nM for 25-OH VD_2_ and 25-OH VD_3_	no report	[64]
5 min	Liquid Chromatography with Tandem-Mass Spectrometry	Prontosil C_18_ analytical column (3 × 250 mm, 3 μm)	0.2% (*v*/*v*) formic acid. Isocratic	13 ng/mL for AA and 11 ng/mL for DHAA	44 ng/mL for ascorbic acid (AA) and 38 ng/mL for dehydroascorbic acid (DHAA)	[65]
30 min	HPLC-MS/MS	ACE-100 C_18_ (2.1 × 100 mm, 3 μm)	A: 10 mM ammonium acetate solution (pH 4.5); B: MeOH with 0.1% acetic acid; C: MeOH with 0.3% acetic acid. Gradient	0.07–170 ng/mL	0.2–520 ng/mL	[66]
12 min	HPLC-MS/MS	HSS T_3_ (2.1 × 150 mm, 1.7 μm)	A: 2 mM ammonium formate in water; B: 2 mM ammonium formate in methanol. Gradient	0.02 μg/100 g	0.12 μg/100 g	[67]
30 min	LC-DAD-MS/MS	a Supelcosil C_18_ (4.6 mm × 50 mm, 5 μm) and an Alltima C_18_ (4.6 mm × 250 mm, 5 μm) for fat-soluble vitamins, ProntoSIL C_30_ column (4.6 × 250 mm, 3 μm) for carotenoids	A: Methanol; B: isopropanol/hexane (50:50, *v*/*v*). Gradient	0.9–15.6 μg/L	2.7–46.8 μg/L	[68]
45 min	LC-MS/MS	Cadenza CD-C_18_ stationary phase (4.6 × 250 mm, 3 μm particles)	A: 20 mM ammonium formate (pH 4.0); B: methanol. Gradient	no report	no report	[69]
8 min	UPLC-MS/MS	HSS T_3_ column (2.1 × 150 mm, 1.8 μm)	A: 0.1% of formic acid in water; B: 0.1% of formic acid in acetonitrile. Gradient	0.06–0.45 μg/100 g	0.12–0.91 μg/100 g	[70]
12 min	HPLC-MS/MS	Pro C_18_ RS column (2.0 × 150 mm, 5 μm)	Methanol–10 mM ammonium formate containing 5 mM methylamine. Isocratic	1.5 ng/mL	3 ng/mL	[71]
24min	UPLC-MS/MS	Alltima C_18_ column (2.1 × 150 mm, 3 μm)	Methanol acidified with 0.1% formic acid. Isocratic	14 ng/L	36 ng/L	[72]
4 min	LC-MS/MS	MAX-RP (2.0 × 50 mm, 4 μm) column	A: 85% methanol, B: 15% ammonium acetate. Isocratic	10 nmol/L	no report	[73]
27 min	LC-MS/MS	HydroRP (2.0 × 250 mm, 4 um) column	A: 0.1% formic acid in water; B: 0.1% formic acid in acetonitrile. Gradient	no report	no report	[74]
24 min	LC-LIT-MS	Polaris C_18_ column (2.1 × 150 mm, 5 μm)	A: Methanol: B: water containing 5 mM ammonium (92:8 *v*/*v*). Isocratic	no report	0.01 μg/100 g	[75]

## Abbreviation

LLE	Liquid–Liquid Extraction
SPE	Solid-Phase Extraction
UAE	Ultrasonic Assisted Extraction
SFE	Supercritical Fluid Extraction
DLLME	Dispersive Liquid–Liquid Microextraction
SDME	Single-Drop Microextraction
HF-LPME	Hollow Fibre Liquid Phase Microextraction
QqLIT	Hybrid Triple Quadrupole-Linear Ion Trap
UPLC or UHPLC	Ultra-High Performance Liquid Chromatography
HPLC	High Performance Liquid Chromatography
SFC	Supercritical Fluid Chromatography
HPLC-MS/MS	High-Performance Liquid Chromatography-Tandem Mass Spectrometry
UV	Ultraviolet
PDA	Photodiode Array
DAD	Diode Array Detector
AA	Ascorbic Acid
ELISA	Enzyme-Linked Immunosorbent Assays
TLC	Thin-Layer Chromatography
CE	Capillary Electrophoresis
TOF	Time of Flight Mass Spectrometry
MEKC	Micellar Electrokinetic Chromatography
